# Targeted therapies for patients with advanced NSCLC harboring wild-type *EGFR*: what’s new and what’s enough

**DOI:** 10.1186/s40880-015-0036-4

**Published:** 2015-07-18

**Authors:** Fei Zhou, Cai-Cun Zhou

**Affiliations:** Department of Medical Oncology, Shanghai Pulmonary Hospital, Tongji University School of Medicine, Shanghai, 200433 P. R. China

**Keywords:** Epidermal growth factor receptor (EGRF), *EGFR* mutation, Anaplastic lymphoma kinase (ALK), *ALK* rearrangement, Molecular targeted therapy, Non-small cell lung cancer

## Abstract

Historically, non-small cell lung cancer (NSCLC) is divided into squamous and nonsquamous subtypes based on histologic features. With a growing number of oncogenic drivers being identified in squamous and nonsquamous NSCLC, this malignancy has been recently divided into several distinct subtypes according to the specific molecular alterations. This new paradigm has substantially highlighted the treatment of advanced NSCLC, shifting it from standard chemotherapy according to specific histologic subtypes to targeted therapy according to specific oncogenic drivers. The application of epidermal growth factor receptor (EGFR)-tyrosine kinase inhibitors (TKIs) in NSCLC patients harboring activating *EGFR* mutations has been a representative model of precise medicine in the treatment of NSCLC. As the role of EGFR-TKIs in routine management of patients with advanced NSCLC has been well established, this review provides an overview of alternative targeted therapy in the treatment of NSCLC, including EGFR-TKIs for patients with wild-type *EGFR* NSCLC, as well as other targeted agents either clinical available or in early- to late-stage development.

## Background

Lung cancer remains the leading cause of cancer-related deaths worldwide, of which non-small cell lung cancer (NSCLC) is the most frequent type [1–3]. The majority of patients with NSCLC have locally advanced or metastatic disease at the time of diagnosis. For a long period, chemotherapy have served as the only backbone of therapeutic strategy for patients with this malignancy, of whom the prognosis is very poor, with a median survival time of only 8–10 months and 5-year survival rate less than 20% [4, 5].

Over the past decade, a great effort has been made regarding the understanding of cancer biology and molecular genetics of NSCLC, and we have witnessed tremendous advances in the management of patients with advanced NSCLC. Based on the presence of specific molecular alterations (oncogenic drivers), NSCLC has been recently divided into several distinct subtypes.

Among the emerging driver oncogenes, epidermal growth factor receptor (*EGFR*) mutation is one of the most important molecular aberrations in patients with NSCLC. Numerous clinical trials have documented the striking efficacy of *EGFR*-tyrosine kinase inhibitors (EGFR-TKIs), namely erlotinib, gefitinib, and afatinib, in advanced NSCLC patients with activating *EGFR* mutations [6–13]. As compared with standard chemotherapeutic regimen, EGFR-TKIs significantly improve objective response rate (ORR), progression-free survival (PFS), and quality of life (QoL) and show mild toxicity. In light of the remarkable progress highlighted by the use of EGFR-TKIs, the treatment of NSCLC has stepped into an era of targeted therapy and precise medicine.

In current clinical practice, it is standard to analyze *EGFR* mutation status in patients with advanced NSCLC when diagnosed. For NSCLC patients harboring activating *EGFR* mutations, EGFR-TKIs are recommended in first-line treatment paradigm. Notably, absence of activating *EGFR* mutation does not imply that chemotherapy remains the only option for patients with wild-type *EGFR* NSCLC. It is intriguing that even in patients with wild-type *EGFR* NSCLC, a considerable proportion of patients may still achieve clinical benefit from EGFR-TKI treatment. Moreover, recent advances in the development of molecular classification of NSCLC have revealed that the majority of driver oncogenes in NSCLC are mutually exclusive of other genetic abnormalities. Therefore, further molecular analysis of wild-type *EGFR* NSCLC might identify additional driver oncogenes (i.e., anaplastic lymphoma kinase [*ALK*], c-ros oncogene 1 [*ROS1*] or rearranged during transfection [*RET*] rearrangement, proto-oncogene protein c-met [*MET*] amplification, human epidermal growth factor receptor 2 [*HER2*] mutation or amplification, v-Raf murine sarcoma viral oncogene homolog B1 [*BRAF*] mutation, fibroblast growth factor receptor 1 [*FGFR1*] amplifications, and discoidin domain receptor-2 [*DDR2*] mutations) and provide alternative targeted therapies.

As the role of EGFR-TKIs in routine management of advanced NSCLC patients harboring activating *EGFR* mutations have been well established, this review focuses on alternative targeted therapy in the treatment of NSCLC, including EGFR-TKIs for patients with wild-type *EGFR* NSCLC. Other targeted agents either clinical available or in early- to late-stage clinical trials will also be discussed.

## EGFR-TKIs in patients with wild-type *EGFR* NSCLC

Clear evidence has demonstrated that EGFR-TKIs should not be used in first-line treatment paradigm in patients with wild-type *EGFR* NSCLC [14]. In the landmark BR.21 study [15], erlotinib significantly prolonged both PFS (2.2 versus 1.8 months; hazard ratio [HR], 0.61; 95% confidence interval [CI], 0.51–0.74; *P* < 0.001) and overall survival (OS, 6.7 versus 4.7 months; HR, 0.70; 95% CI, 0.58–0.85; *P* < 0.001) compared with placebo in previously treated patients with advanced NSCLC. The INTEREST [16] and TITAN [17] studies further demonstrated that EGFR-TKIs are not inferior standard second-line chemotherapy (docetaxel or pemetrexed) for unselected NSCLC patients. What is the role of EGFR-TKIs as second-line therapy for patients with wild-type *EGFR* NSCLC?

The TAILOR study addressed this issue. In TAILOR study [18], patients assigned to the chemotherapy arm (docetaxel) experienced a statistically significant improvement in PFS (2.9 versus 2.4 months; HR, 0.71; 95% CI, 0.53–0.92; *P* = 0.02) and OS (8.2 versus 5.4 months; HR, 0.73; 95% CI, 0.53–1.00; *P* = 0.05) compared with erlotinib in second-line therapy for patients with advanced wild-type *EGFR* NSCLC. The DELTA study and CTONG0806 studies consistently supported the conclusions of the TAILOR study. In DELTA study [19], erlotinib was significantly inferior to docetaxel in terms of PFS (1.3 versus 2.9 months; HR, 1.45; 95% CI, 1.09–1.94; *P* = 0.01) and ORR (5.6% versus 20.0%, *P* < 0.01) in patients with wild-type *EGFR* NSCLC. In CTONG0806 study [20], patients assigned to the chemotherapy arm (pemetrexed) experienced a statistically significant improvement in PFS (4.8 versus 1.6 months; HR, 0.54; 95% CI, 0.40–0.75; *P* < 0.001) and a trend toward improvement in OS (12.4 versus 9.6 months; HR, 0.72, 95% CI 0.49–1.04; *P* = 0.077) compared with gefitinib in second-line therapy for patients with advanced nonsquamous NSCLC harboring wild-type *EGFR* mutations (Table 1). Moreover, a recent meta-analysis including 1,605 patients with wild-type *EGFR* NSCLC in 11 trials demonstrated that chemotherapy showed a superiority in terms of PFS (HR, 1.84; 95% CI, 1.35–2.52) and ORR (16.8 versus 7.2%; relative risk, 1.11; 95% CI, 1.02–1.21) compared with EGFR-TKIs [21].

**Table 1:** EGFR-TKIs versus chemotherapy as second-line treatment for advanced NSCLC patients harboring wild-type *EGFR* mutations

Trial	Detection technique	Treatment	PFS (months)	HR for PFS (95% CI)	OS (months)	HR for OS (95% CI)	Reference
TAILOR	Sequencing	Erlotinib	2.4	0.71 (0.53–0.95)	5.4	0.73 (0.53–1.00)	[18]
		Docetaxel	2.9		8.2		
DELTA	PCR-based methods	Erlotinib	1.3	1.45 (1.09–1.94)	9.0	0.98 (0.69–1.39)	[19]
		Docetaxel	2.9		10.1		
CTONG0806	DNA sequencing	Gefitinib	1.7	0.53 (0.38–0.75)	9.6	0.72 (0.49–1.04)	[20]
	ARMS	Pemetrexed	5.6		12.4		

*EGFR* epidermal growth factor receptor, *TKIs* tyrosine kinase inhibitors, *NSCLC* non-small cell lung cancer, *PCR* polymerase chain reaction, *ARMS* Scorpion amplification refractory mutation system, *PFS* progression-free survival, *HR* hazard ratio, *CI* confidence interval, *OS* overall survival.

Do these results mean that EGFR-TKIs should be absolutely banned in patients with wild-type *EGFR* NSCLC? Not necessary. In clinical practice, a considerable proportion of patients with wild-type *EGFR* NSCLC may achieve clinical benefit from EGFR-TKIs. Therefore, to discover the potential mechanism or to identify applicable population who may benefit from EGFR-TKIs is of clinical value, especially for patients who do not have specific molecular alterations. The clinically validated, serum-based protein test called VeriStrat (Biodesix, Broomfield, CO, USA) may be a promising strategy to achieve this goal. The PROSE study was to prospectively evaluate the predictive utility of VerStrat on the survival of NSCLC patients treated with second-line erlotinib or chemotherapy [22]. Patients were classified into Good and Poor groups based on VerStrat. In VerStrat Good group of NSCLC patients with wild-type or unknown status *EGFR*, the OS was not different between the patients treated with chemotherapy and those treated with erlotinib (10.9 versus 11.0 months; HR, 1.06; 95% CI, 0.77–1.46; *P* = 0.714), suggesting that EGFR-TKIs could be a rational choice for this subpopulation in second-line setting. Recently, Li et al. [23] reported that patients with higher microRNA-200c expression achieved longer PFS (5.0 versus 1.2 months; HR, 0.38; 95% CI, 0.21–0.70, *P* = 0.002) and OS (9.6 versus 5.0 months; HR, 0.54; 95% CI, 0.30–0.96, *P* = 0.035) than those with lower microRNA-200c expression in patients with wild-type *EGFR* NSCLC. Ren et al. [24] more recently found that patients with epithelial phenotype responded better to EGFR-TKIs than those with epithelial-to-mesenchymal or mesenchymal phenotype in patients with wild-type *EGFR* NSCLC in terms of PFS (4.4 versus 1.9 versus 1.0 months, *P* < 0.001) and OS (11.5 versus 8.9 versus 4.9 months, *P* < 0.001). In a recent study by Toffalorio et al. [25], among patients with wild-type *EGFR* NSCLC, 13 patients with high polysomy of chromosome 7 received erlotinib, in which the disease control rate was as high as 76.9% (1 patient with complete response, 4 patients with partial response, and 5 patients with stable disease); the mean time-to-progression in this subpopulation was 9 months, suggesting a potential role of high polysomy of chromosome 7 as a useful biomarker to identify patients harboring wild-type *EGFR* mutations who may benefit from EGFR-TKIs. The survival in aforementioned subpopulations, namely wild-type *EGFR* NSCLC patients with higher microRNA-200c expression, epithelial phenotype, or high polysomy of chromosome 7 is promising, and it deserves further investigation in these subpopulations.

## *EGFR* mutation detection methods

Interestingly, in the CTONG0806 study [20], compared with Scorpion amplification refractory mutation system (ARMS) method, false negative rate of DNA sequencing was 29.6% (32/108). The ORR of patients treated with gefitinib in ARMS mutation-positive group was higher than that in ARMS mutation-negative group (38.5 versus 10.5%, *P* = 0.09). Consequently, the results of CTONG0806 study raised a controversial issue in clinical practice: which method should be used to detect *EGFR* mutations?

Among a variety of methods used for *EGFR* mutation genotyping, Sanger sequencing has been the most widely used method, which is often considered the ‘‘gold standard’’ for *EGFR* mutation testing [26]. However, Sanger sequencing is recommended when the percentage of tumor cell contents in the sample is at least 50% [27]. Hence, Sanger sequencing may offer limited sensitivity, and false negative results can occur more frequently in small biopsy samples without sufficient tumor cell contents or high-quality DNA. Immunohistochemistry (IHC) assay using commercially available mutation-specific rabbit monoclonal antibodies directly against two major forms of *EGFR* mutations, namely deletions in exon 19 and L858R point mutation, demonstrated a high concordance with Sanger sequencing, with an excellent sensitivity of 92% and specificity of 99% [28]. Although mutation-specific IHC has been demonstrated to be a rapid, sensitive, and cost-effective method for detecting the two predefined *EGFR* mutations [29–32], even in small bronchial biopsy samples [33], other uncommon *EGFR* mutations, including T790M mutations, cannot be detected by the mutation-specific antibodies; it is currently lack of sufficient data to make an evidence-based recommendation for the use of IHC assay for *EGFR* mutation detection [27, 34]. A number of polymerase chain reaction (PCR)-based assays have also been employed for *EGFR* mutation testing, including ARMS (DxS, Manchester, UK) [35], cationic conjugated polymer (CCP)-based fluorescence resonance energy transfer (FRET) [36], peptide nucleic acid-locked nucleic acid (PNA-LNA) PCR clamp (Stratagene, La Jolla, CA, USA) [37], denaturing high-performance liquid chromatography (HPLC) (Transgenomic, Omaha, NE, USA) [38], MassARRAY System (Sequenom Inc., San Diego, CA, USA) [39], SNaPshot assay (Applied Biosystems, Life Technologies, Foster, CA, USA) [40, 41], and TaqMan Mutation Detection Assay (Applied Biosystems, Life Technologies, Foster, CA, USA) [42]. Compared with Sanger sequencing, the PCR-based assays are rapid and more sensitive and have ability to detect a mutant *EGFR* sequence in small biopsy specimens containing less that 10% mutated DNA, including cell block samples [43]. The main theoretical drawback of these PCR-based assays is their inability to detect some of the rare *EGFR* mutations which are detectable by direct sequencing [44].

As mentioned above, each method for *EGFR* mutation testing has its own strengths and limitations. In general, for samples with high tumor cell contents, the majority genotyping methods are suitable; however, for samples without sufficient tumor cell contents, more sensitive methods are preferable. Furthermore, the choice of method in clinical practice should be made based on the testing laboratory’s expertise, detection instrument, and the detection purpose.

Coming back to our topic, which method should be used to confirm a “true” wild-type *EGFR* mutation? Notably, patients with a low abundance of *EGFR* mutations (ARMS positive but sequencing negative) achieved more clinical benefits from EGFR-TKIs than those with wild-type *EGFR* NSCLC (both ARMS and sequence negative) with respect to both PFS and OS [45]. Similar survival benefit trend was also observed in the CTONG0806 study [20]. Since patients with a low abundance of *EGFR* mutations may achieve more clinical benefits from EGFR-TKI treatment, more sensitive and advanced assays, such as droplet-digital PCR (next-generation quantitative PCR) or next-generation sequencing [46, 47], may be a preferable option to more accurately evaluate *EGFR* mutation status and confirm “true” wild-type *EGFR* NSCLC.

## *ALK* rearrangements

The application of *ALK* inhibitors has been another representative model of targeted therapy in the treatment of NSCLC. *ALK* gene rearrangements were first identified in NSCLC in 2007 [48]. Crizotinib, an *ALK/ROS1/MET* multi-targeted TKI [49], has been approved by the United State Food and Drug Administration (FDA) only 4 years after the first report of *ALK* rearrangement in NSCLC. *ALK* rearrangements occur in approximately 3–5% of NSCLC patients [50–52], more common in patients with adenocarcinoma, younger patients, and never or light smokers as well as generally mutually exclusive with other identified oncogenic drivers. It was reported that the frequency of *ALK* rearrangement was as high as 33% in never or light smokers without *EGFR* mutations [50]. Hence, *ALK* rearrangements represent another distinct molecular subtype of NSCLC and serve as a novel molecular target in NSCLC, especially for patients who do not harbor activating *EGFR* mutations.

At present, the role of crizotinib in *ALK*-rearranged NSCLC as second- and first-line settings has been well established in two international, randomized phase III studies [53, 54]. The PROFILE 1007 was a phase III study comparing crizotinib with docetaxel or pemetrexed in *ALK*-rearranged NSCLC as second-line setting [53]. In the PROFILE 1007 study, crizotinib yielded a significant improvement in PFS (7.7 versus 3.0 months; HR, 0.49; 95% CI, 0.37–0.64; *P* < 0.001), ORR (65 versus 20%, *P* < 0.001), and global QoL as compared with chemotherapy [53]. Subsequent PROFILE 1014 study further confirmed the striking efficacy of crizotinib in *ALK*-rearranged NSCLC as first-line treatment [54]. In the PROFILE 1014 study, 343 patients with *ALK*-rearranged nonsquamous NSCLC were randomly assigned to receive crizotinb (172 patients) or cisplatin plus pemetrexed (171 patients), the most effective chemotherapy regimen in patients with nonsquamous NSCLC. Again, crizotinib demonstrated a superiority over chemotherapy in PFS (10.9 versus 7.0 months; HR, 0.45; 95% CI, 0.35–0.60; *P* < 0.001) and ORR (74 versus 45%, *P* < 0.001) (Table 2) [54]. In current clinical practice [55], crizotinib has been a standard care for patients with *ALK*-rearranged NSCLC, and *ALK* rearrangement should be routinely analyzed in patients with advanced NSCLC, particularly for patients with wild-type *EGFR* NSCLC.

Ceritinib (LDK378) is a selective, potent, next-generation *ALK* inhibitor. In a phase I study (ASCEND-1) [56], ceritinib showed promising efficacy in patients with *ALK*-rearranged NSCLC who had been treated with crizotinib previously and who were crizotinib naïve. For patients who were crizotinib naive and treated with at least 400 mg of ceritinib daily, the ORR was 62%. Among patients who had previously received crizotinib, the ORR was 56% (95% CI, 45–67%). The median PFS was 7.0 months in the entire population, 6.9 months in the subgroup of patients who had progressed on crizotinib previously, and 10.4 months in the subgroup of patients who had not received crizotinib previously (Table 2). In contrast to crizotinib, ceritinib also showed activity in patients with brain metastases. The median PFS was 6.9 months in patients with central nervous system (CNS) metastases at baseline, which was similar to that in patients without CNS metastases (7.0 months). In light of the striking results of this phase I study, an expansion cohort trial including 180 *ALK*-rearranged NSCLC receiving ceritinib at the recommended dose (750 mg/day) has been reported at the 2014 American Society of Clinical Oncology (ASCO) Annual Meeting and the results were promising [57].

Alectinib is another potent, second-generation *ALK* inhibitor. In a phase 1/2, single-arm, open-labelled study (AF-001JP) [58], 93.5% (43/46) of patients with *ALK*-rearranged NSCLC who had not received crizotinib previously achieved an objective response when receiving alectinib at the recommended dose (300 mg twice per day). Preclinical studies have demonstrated that alectinib is also active against crizotinib-resistant secondary *ALK* mutations (including L1196M, C1156Y, and F1174L) [59]. In a more recent phase 1/2 study (AF-002JG) [60], alectinib showed remarkably efficacy in patients with *ALK*-rearranged NSCLC who had progressed on or were intolerant to crizotinib, with an ORR of 55% (24/44) (Table 2). Among 21 patients with CNS metastases at baseline, 11 (52%) had an objective response.

**Table 2:** Published or presented studies with results in NSCLC patients treated with targeted agents beyond EGFR-TKIs

Target	Agent	Phase	Eligibility	Number of patients	ORR (%)	PFS (months)	OS (months)	References
*ALK*	Crizotinib	III	Pretreated, *ALK*-rearranged	347	65	7.7	20.3	[53]
	Crizotinib	III	Untreated, *ALK*-rearranged	343	74	10.9	NR	[54]
	Ceritinib	I	Pretreated, *ALK*-rearranged	114 (at least 400 mg)	58 (overall population)	7.0	NR	[56]
					56 (crizotinib-treated)	6.9		
					62 (crizotinib-naive)	10.4		
	Alectinib	I–II	*ALK*-rearranged	46	93.5	NR	NR	[58]
			*ALK* inhibitor-naive					
	Alectinib	I–II	*ALK*-rearranged, crizotinib-resistant	47	55	NR	NR	[60]
*ROS1*	Crizotinib	I	*ROS1*-rearranged	50	72	19.2	NR	[61]
*MET*	Crizotinib	I	*MET*-amplified	13	20 (intermediate amplification)	NR	NR	[62]
					50 (high amplification)			
*BRAF*	Dabrafenib	II	*BRAF* ^*V600E*^ mutation-positive	17	54	NR	NR	[63]
*HER2*	Anti-HER2 agents	Retrospective	*HER2* mutation-positive	16	50	5.1	NR	[64]
*FGFR1*	BGJ398	I	*FGFR1*-amplified	17	11.7	NR	NR	[65]

*ALK* anaplastic lymphoma kinase, *ROS1* c-ros oncogene 1, *MET* proto-oncogene protein c-met, *BRAF* v-Raf murine sarcoma viral oncogene homolog B1, *HER2* human epidermal growth factor receptor 2, *FGFR1* fibroblast growth factor receptor 1, *ORR* objective response rate, *NR* not reported or not reached. Other abbreviations as in Table 1.

The question of timing, preferable choice, and sequence of various *ALK* inhibitors in patients with *ALK*-rearranged NSCLC has not yet been well answered to date. In a more recently retrospective study [66], sequential treatment with crizotinib and ceritinib yielded an impressive survival in *ALK*-rearranged NSCLC, with a PFS of 17.4 months and an OS of 49.4 months. Knowledge regarding the choice of *ALK* inhibitors will also be expanded from an ongoing, randomized phase 3 trial (NCT02075840), head-to-head comparing alectinib with crizotinib in previously untreated patients with advanced *ALK*-rearranged NSCLC.

## *ROS1* rearrangements

*ROS1* rearrangements define another distinct molecular subtype of NSCLC and have been identified as a novel oncogenic driver in the targeted therapy of NSCLC [67]. Similar to the clinical features of *ALK*-rearranged NSCLC, *ROS1* rearrangement are more likely to be found in younger, never smokers with histologic diagnosis of adenocarcinoma, with an occurrence rate ranging from 1 to 2% [67–69]. In preclinical studies, crizotinib was active in cell lines harboring *ROS1* rearrangement [67, 70]. In a phase I study [61], 50 patients with *ROS1*-rearranged NSCLC were treated with crizotinib at the standard oral dose of 250 mg twice daily. The ORR was 72% (95% CI, 58–84%), and median PFS was 19.2 months (95% CI, 14.4 months to not reached) (Table 2). Several phase II trials are underway to evaluate the safety and efficacy of crizotinib in advanced *ROS1*-rearranged NSCLC in East Asian patient population (NCT01945021) and in European patient population (NCT02183870). An open-labelled, multicenter, phase II trial assessing the efficacy of ceritinib (LDK378) in patients with *ROS1*-rearranged NSCLC is also ongoing (NCT01964157).

## *MET* amplification

*MET* gene, the only known receptor for hepatocyte growth factor, has been shown to be associated with acquired resistance to EGFR-TKIs in NSCLC patients [71, 72]. Because the MET signaling pathway could crosstalk with other signaling receptors and *MET* amplification could be concomitant with other oncogenic drivers, the role of de novo *MET* amplification as primary oncogenic driver remains controversial in NSCLC. Recently, dramatic response to crizotinib in patients with de novo *MET* amplification was observed in several case reports [73, 74]. Notably, the reported patients were both absence of other known oncogenic drivers, such as *EGFR* mutations and *ALK/ROS1* rearrangement, suggesting that de novo *MET* amplification might be a primary oncogenic driver in a subtype of NSCLC. In a more recent phase I study [62], crizotinib showed antitumor activity in patients with *MET* amplification, especially in those with high *MET* amplification, with an ORR of 50% (3/6) (Table 2). Several small-molecule inhibitor of *MET*, such as ARQ197 (NCT01244191, NCT01395758, and NCT00777309) and XL184 (NCT00596648), are also being evaluated in advanced NSCLC; however, the enrolled patients are not selected based on *MET* expression.

## *RET* rearrangements

*RET* is a receptor tyrosine kinase that plays an important role in cell proliferation, neuronal navigation, cell migration, and cell differentiation [75]. Recently, *RET* rearrangements have been demonstrated to be a novel driver oncogene in a subtype of NSCLC [76]. In contrast to thyroid cancer, in which *RET* rearrangement is one of the most common molecular alterations and can be found in up to 80% of tumors [77], *RET* rearrangements are present only in 1–2% of patients with NSCLC [76, 78–81]. *RET* rearrangements tended to occur in patients who were younger than 60 years, never-smokers, with early lymph node metastases, with poorly differentiated tumors, and with a solid-predominant subtype of tumor [76]. Vandetanib, a multi-tyrosine kinase inhibitor that inhibits vascular endothelial growth factor receptor (*VEGFR*), *EGFR*, and *RET,* showed dramatic efficacy in a patient with *RET* rearrangement [82]. In contrast to previous studies that evaluated the role of vandetanib in unselected NSCLC, a phase II study is ongoing to investigate the efficacy and safety of vandetanib in patients with advanced NSCLC with *RET* rearrangements (NCT01823068). Cabozantinib, also a multi-kinase inhibitor and potent inhibitor of *RET*, demonstrated impressive activity in patients with *RET* fusion-positive lung adenocarcinoma [83]. The efficacy of cabozantinib in patients with advanced *RET* fusion-positive NSCLC is prospectively being evaluated in a phase II study (NCT01639508).

## *BRAF* mutations

*BRAF* belongs to the family of *RAF* kinases, which are intracellular effectors of the mitogen-activated protein kinase (MAPK) signaling cascade [84]. *BRAF* mutations are generally mutually exclusive of *EGFR* mutations and proto-oncogene protein p21 (c-Ki-ras) (*KRAS*) mutations. In a retrospective analysis of 1,046 NSCLC patients in Caucasian population, *BRAF* mutations were present in 4.9% (36/739) of lung adenocarcinoma and 0.3% (1/307) of squamous cell carcinoma (SqCC) [85]. V600E *BRAF* mutation, a domain subtype of *BRAF* mutations, was significantly more common in females and was identified in 8.6% of female patients with adenocarcinoma, which is helpful to identify the enriched patient population for treatment with *BRAF* inhibitors. Dabrafenib is a potent and selective inhibitor of *BRAF* kinase activity. In interim analysis of a single-arm, phase II study, 17 pretreated NSCLC patients carrying V600E *BARF* mutations were treated with dabrafenib [63]. The results were encouraging, with an ORR of 54% (7 patients with partial response) (Table 2). Dramatic response was also observed in several case reports of NSCLC patients harboring activating V600E *BRAF* mutations treated with vemurafenib, another inhibitor of *BRAF* kinase [86–88].

## *HER2* mutations

Similar to *EGFR*, *HER2* is also a member of the *ErbB* family of receptor tyrosine kinases. HER2-targeted agents, such as trastuzumab, pertuzumab, and lapatinib, represent a successful use of targeted therapy for breast cancer with *HER2* overexpression or amplification. However, these agents failed to demonstrate significant improvement in survival of NSCLC patients with *HER2* overexpression when administered as monotherapy or in combination with chemotherapy [89–91]. Notably, several retrospective studies suggested that NSCLC patients with positive *HER2* mutations may benefit from HER2-targeted therapy [64, 92]. In a large retrospective study including 3,800 NSCLC patients, *HER2* mutations were present in 65 patients (1.7%) [64]. In 16 patients receiving subsequent 22 anti-HER2 therapies, 11 patients achieved partial response, with an ORR of 50% and a disease control rate of 82%. The PFS was also encouraging, as long as 5.1 months (Table 2). However, as aforementioned, *HER2* mutation is a rare event in lung cancer, with a prevalence of <2%, a little more frequently in those who are never smokers, adenocarcinoma patients, and females [64, 92, 93].

## *FGFR1* amplifications

The *FGFR* family of tyrosine kinase receptors comprises 4 highly conserved kinases (*FGFR1*-*4*) and plays a pivotal role in cancer cell proliferation, migration, angiogenesis, and patient survival [94]. Among the 4 members, *FGFR1* amplifications seem to exist exclusively in SqCC, with a presence of about 19%, more common in smokers and patients with lymph node metastasis [95]. Preliminary studies have demonstrated that focal *FGFR1* amplification was associated with response to treatment with *FGFR* inhibitors [96, 97]. In a phase I dose-escalation study, 21 patients with *FGFR1*-amplified SqCC received treatment with a selective pan-FGFR inhibitor (BGJ398) [65]. Among 17 evaluable patients at data cutoff, 2 patients achieved partial response and 3 patients had stable disease (Table 2). A prospective clinical trial assessing the efficacy of ponatinib (a multi-kinase *FGFR* inhibitor) on advanced SqCC with *FGFR* kinase alterations is also underway (NCT01761747).

## *DDR2* mutations

The *DDR2* is a receptor tyrosine kinase, which acts as a collagen receptor and plays a role in cell migration, proliferation, and survival [98]. A preliminary study demonstrated that *DDR2* mutations were present in 3.8% of patients with SqCC and that *DDR2* mutations were associated with response to dasatinib, a multi-kinase inhibitor that inhibits *DDR1* and *DDR2* [99], suggesting *DDR2* mutations may be primary oncogenic driver in patients with SqCC. A phase II study is designed to evaluate the efficacy of dasatinib as first- or subsequent-line therapy in SqCC patients harboring *DDR2* mutations (NCT01514864). However, this trial has been terminated due to the lack of efficacy and slow accrual.

## Conclusions and perspectives

Historically, NSCLC is divided into squamous and nonsqumous types based on histologic features. With a growing number of new oncogenic drivers being identified in squamous and nonsqumous NSCLC, this malignancy has been divided into several distinct subtypes according to the specific molecular alterations. The tremendous advances of EGFR-TKIs represent a success of precise medicine in the treatment of NSCLC. Afterwards, this treatment paradigm of NSCLC has been substantially shifted. In current clinical practice, when a new NSCLC patient is diagnosed, every effort should be made to obtain a tumor sample for genotyping. For patients who have specific oncogenic alterations, matching targeted therapy would be a preferable treatment option. Notably, absence of *EGFR* mutations does not imply that classical chemotherapy remains the only treatment option. Publications also provide evidence to select appropriate patient population who might benefit from EGFR-TKIs even in patients with wild-type *EGFR* NSCLC. More importantly, because the majority of driver oncogenes in NSCLC are mutually exclusive of other genetic abnormalities, further molecular analysis of wild-type *EGFR* NSCLC might identify additional driver oncogenes and provide alternative targeted therapies. In addition, with the help of multiplex genotyping technique [100], it is being able to simultaneously identify multiple genes from one tumor sample. Therefore, we may not be entangled in deciding which oncogenic driver to detect in the near future.

On the other hand, identification of therapeutic targets for SqCC has lagged behind the advances in lung adenocarcinoma. Although plenty of the somatic molecular alterations have been identified in SqCC, targeted therapy for molecularly defined subtypes of SqCC according to specific oncogenic drivers is still underway. Encouragingly, recent advances in the development of immune therapy that blocks the immune checkpoints [101, 102], including programmed death-1 (PD-1) and programmed death-ligand 1/2 (PD-L1/2), highlight the essential role of immune therapy in the treatment of NSCLC in the future, especially for patients with SqCC [103].
